# How Do We Measure Stress in Latinos in the United States? A Systematic Review

**DOI:** 10.1089/heq.2020.0112

**Published:** 2021-05-19

**Authors:** Shanna D. Stryker, Robert Andrew Yockey, Julia Rabin, Lisa M. Vaughn, Farrah Jacquez

**Affiliations:** ^1^ Department of Family and Community Medicine, University of Cincinnati College of Medicine, Cincinnati, Ohio, USA.; ^2^ School of Human Services, College of Education, Criminal Justice, and Human Services, University of Cincinnati, Cincinnati, Ohio, USA.; ^3^ Department of Psychology, College of Arts and Sciences, University of Cincinnati, Cincinnati, Ohio, USA.; ^4^ Department of Pediatrics, Cincinnati Children's Hospital Medical Center/University of Cincinnati College of Medicine, Cincinnati, Ohio, USA.

**Keywords:** Latino, Hispanic, stress, review, psychometric, Latinx

## Abstract

**Background:** Previous research has documented that Latinos report higher levels of stress than other ethnicities and are an increasing portion of the demographics of the United States. While there are many measures to assess stress and other stress-related conditions, there are no systematic reviews to date to assess whether the current measures of generalized stress are valid or reliable in Latinos in the United States. The purpose of this systematic review was to examine the current state of the literature assessing the psychometric properties in stress measures in this population.

**Methods:** We used Preferred Reporting Items for Systematic Reviews and Meta-Analyses (PRISMA) guidelines to review the literature from January 1990 to May 2020 for studies, which measured the psychometric properties of scales measuring generalized stress in Latinos in the United States.

**Results:** Twelve studies measured the psychometric properties of eight scales of generalized stress. The 10-item Perceived Stress Scale, the Hispanic Stress Inventory, the Hispanic Women's Social Stressor Scale, and the Family Obligation Stress Scale show the strongest reliability and validity for measuring stress in Latinos in the United States. Most studies were done in traditional immigration destinations in the United States.

**Conclusion:** While four scales which show acceptable reliability and validity for measuring stress in Latinos in the United States, continuing to develop and further validate these scales within Latino communities will be critical to understand and address Latino stress more comprehensively. Our findings can inform health research and clinical interventions for this at-risk community.

## Introduction

Latinos are a rapidly growing demographic group in the United States, accounting for more than half of the population growth in the country between 2010 and 2019,^1^ and report higher levels of stress than any other ethnic group.^2,3^ Stress can affect both physical and mental health and be detrimental to health behaviors such as eating and sleeping. There are associations between stress and an increased prevalence of, and worse outcomes for, depression, HIV/AIDS, cancer, gastrointestinal and endocrinologic health, and cardiovascular disease.^4,5^ Adults in the United States are increasingly recognizing the negative impact of stress on their health and health behaviors,^2^ concern over the impact of stress on one's own health has independently been found to be detrimental to overall health^6^ and cardiovascular health.^7^

The link between stress and health has been well demonstrated in Latinos. A national study (the Hispanic Community Health Study/Study of Latinos [HCHS/SOL], *n*=5,313 for the Sociocultural Ancillary Study) shows higher prevalence of coronary artery disease, stroke, diabetes, and hypertension in Latinos who report chronic stress,^8^ and glucose dysregulation has also been observed related to chronic stress.^9^ Perceived stress has been linked to higher levels of depression and worse general health.^10^ Health behaviors are also impacted: higher levels of stress in Latinos has been associated with lower levels of physical activity,^11^ higher rates of smoking,^8^ and worse sleep.^12^

In addition to exposure to stressors common across ethnicities such as family, finances, work, and health, Latinos in the United States are more likely to experience fear of deportation and ethnic discrimination, which has independently been linked to higher stress levels and worse health.^2,3,10,13–18^ Stress specific to acculturation (cultural assimilation to the prevailing culture of their destination) has been linked to anxiety, depression, and poor metabolic effects in Latino immigrants.^19,20^ Measures of acculturative stress and the impact of acculturative stress on mental health have been well-studied,^21,22^ but there have been no reviews that have described the psychometric properties of scales measuring generalized stress in Latinos in the United States.

The purpose of this systematic review of the literature is to describe the psychometric properties of existing scales measuring generalized stress in Latino adults in the United States. It is critical to be able to more accurately quantify stress in an ethnic group disproportionately affected by higher stress levels so that we can (1) better understand the effects of generalized stress on the health and wellbeing of Latinos, and (2) better measure the impact of stress management interventions on stress levels.

## Methods

We identified measures that assessed stress in US-based Latinos published literature from January 1990 through May 2020. We searched using computer-based literature search engines (e.g., PubMed, Medline and PsychInfo) for our systematic literature review and defined generalized stress as “mental or emotional distress, strain, and/or tension associated with feelings of fatigue and being overwhelmed” when evaluating relevant measures.

**FIG. 1.**
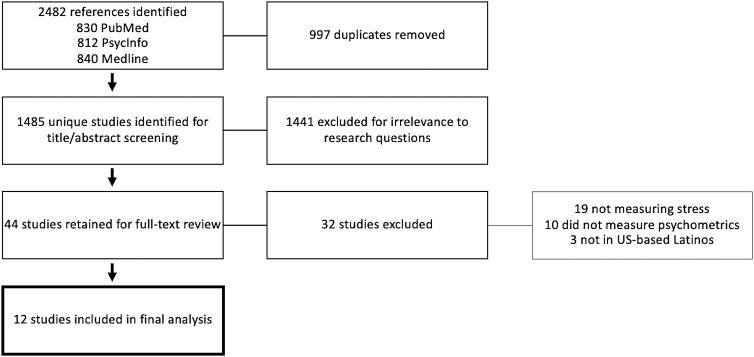
Preferred Reporting Items for Systematic Reviews and Meta-Analyses (PRISMA) flow diagram.

Our article utilized the Preferred Reporting Items for Systematic Reviews and Meta-Analyses (PRISMA) systematic review guidelines (Fig. 1 and Table 1).^23^ Articles were included if they were written in English, measured psychometric properties of scales of generalized stress, and were conducted with Hispanic/Latino populations in the United States. Articles were excluded if they described validation done outside of the United States or if they did not measure the scales in Latino populations, if they focused on validation in children/adolescents, if they measured anything other than generalized emotional stress (such as post-traumatic stress or acculturative stress), and/or did not measure psychometric properties of the scales. We calculated a kappa score for percent agreement and inter-rater reliability using Stata v. 15.1 (*k*=0.87), which demonstrated a strong level of agreement among coders.^24^

**Table 1.** Search Terms Used in Literature Review

1	Stress
2	hispanic OR latin^*^
3	scale OR measure OR questionnaire OR survey OR inventor OR index OR checklist
4	child^*^ OR adolescen^*^
5	posttraumatic OR acculturat^*^
	1 AND 2 AND 3 NOT 4 NOT 5

^*^ Asterisks used when possible to include multiple endings (e.g., latin^*^ would include Latino, Latina, Latinx, Latinos, etc.).

## Results

Twelve studies described the psychometric properties of scales measuring stress in US-based Latinos. The psychometric characteristics of each of these measures are described in Table 2.

**Table 2.** Psychometric Properties of Scales Described in Identified Studies

Study, author (year)^Ref.^	Scale	Language	IC	CNV	CRV	FS	CSV	*n*	Study population notes
Baik et al. (2019)^25^	Perceived Stress Scale-10	Spanish	0.78	?	−	+	+	226	Community sample of Hispanic American adults in southern California
		English	0.87	?	−	+	+	210	
Cavazos-Rehg et al. (2006)^26^	Hispanic Stress Inventory—Immigrant Version, abbreviated version	Spanish and English	0.86–0.87	?	+	+	+	143	Community sample of adult Hispanic immigrants in Missouri
Cervantes et al. (1990)^27^	Hispanic Stress Inventory	Spanish and English	0.77–0.91	+	+	+	+	493	Recent Mexican/Central American immigrants and US-born Mexican-Americans in southern California
Cervantes et al. (1991)^28^	Hispanic Stress Inventory	Spanish and English	0.77–0.91	+	+	+	+	Phase 1: 105; Phase 2: 5; Phase 3: 493; Phase 4: 35	Recent Mexican/Central American immigrants and US-born Mexican-Americans in southern California
D'Alonzo (2011)^29^	Hispanic Stress Inventory—Immigrant Version, revised	Spanish	0.74	+	−	+	+	Phase 1: 13; Phase 2: 6; Phase 3: 81	Community sample of immigrant Latinas in New Jersey
Goodkind et al. (2008)^30^	Hispanic Women's Social Stressor Scale	Spanish and English	0.73–0.94	+	+	+	+	Phase 1: 12; Phase 2: 269	US-born and Mexico-born Hispanic mothers in New Mexico
Hannan et al. (2015)^31^	Life Events Inventory	Spanish and English	0.94–0.97	−	+	−	?	63	Bilingual Hispanic women that were faculty, staff, students or friends of these at a Florida University
	Daily Hassles Scale	Spanish	0.86–0.94	−	+	−	?		
		English	0.92–0.94						
Katerndahl et al. (2002)^32^	Duke Social Support and Stress Scale	English	0.58–0.72	−	−	−	+	80	English-Speaking Hispanic adults from a primary care clinic in Texas
Molina et al. (2019)^33^	Family Obligation Stress	Spanish and English	0.76–0.77	+	−	+	+	Phase 1: 17; Phase 2: 539	Hispanic women ages 42–74 receiving services at specific Washington state clinics
Novy et al. (2001)^34^	Worry Scale	Spanish	0.86–0.96	−	+	−	−	98	Community sample of bilingual Hispanic adults in Texas with an anxiety diagnosis
		English	0.89–0.97						
Perera et al. (2017)^35^	Perceived Stress Scale-10	Spanish	0.84	+	+	+	+	5,313	Hispanic adults recruited from NYC, Chicago, Miami, and San Diego that were part of the Hispanic Community Health Study/Study of Latinos
		English	0.86						
Teresi et al. (2020)^36^	Perceived Stress Scale-10	Spanish and English	0.88	−	−	+	+	453	Community sample of Hispanic (Dominican, Puerto Rican, Mexican) adults that are caregivers for individuals with Alzheimer's disease in New York City

CNV, content validity; CRV, criterion-related validity; CSV, construct validity; FS, factor structure; IR, internal consistency; *n*, sample size.

### Perceived Stress Scale

The 10-item Perceived Stress Scale (PSS-10) is a self-report scale, which measures generalized stress with questions such as “In the last month, how often have you felt confident about your ability to handle your personal problems?”^37^ Three studies in this review measured the psychometric properties of the PSS-10 in Latino populations in the United States, all in both Spanish and English.

Baik et al.^25^ utilized this scale among a community sample of Hispanic American adults and found an internal consistency of alpha=0.78 for the Spanish version and 0.87 for the English version in this sample, but did not measure content validity or criterion-related validity. Conducted with a much larger community sample of Hispanic adults from major metropolitan centers (the HCHS/SOL), Perera et al.^35^ demonstrated a higher internal consistency in their Spanish version of the PSS-10 (alpha=0.84) than did Baik et al. and demonstrated good construct validity, criterion-related validity, and factor structure of the scale. Finally, Teresi et al.^36^ used the same scale to measure the stress of Hispanic caregivers for individuals with Alzheimer's disease and noted good internal consistency (alpha=0.88), but they did not measure content validity or criterion-related validity. None of these studies measured content validity of the scale, and each of these studies was done in locations that are traditional immigration destinations.

### Hispanic Stress Inventory

The original Hispanic Stress Inventory (HSI) has 59 items assessing stress in US-born Hispanic individuals and 73 items assessing stress in Hispanic immigrants.^28^ Respondents indicate whether they have had any of 73 stressful experiences and if so, how worried or tense it made them feel on a 5-point Likert scale. The following subscales are present: Occupation/Economic Stress (“I have been criticized about my work”), Parental Stress (“I have seen my son/daughter behave delinquently”), Marital Stress (“My spouse has not helped with household chores”), Family/Culture Stress (“I have felt that being too close to my family interfered with my own goals”); in the immigrant version there is an additional Immigration Stress subscale (“I have not been able to forget the last few months in my home country”).

Four studies measured the psychometric properties of the HSI in Latino populations based in the United States. The original full scale, as discussed by Cervantes et al.,^27,28^ was developed in southern California and the subscales showed good internal consistency (alpha=0.77–0.91). Content, criterion-related, and construct validity were all measured, as well as factor structure in the original study and was found to have good to excellent reliability and validity. D'Alonzo measured a revised version of the Immigrant version of the HSI among a diverse group of Latinos in New Jersey and found an internal consistency of alpha=0.74 and measured both content and construct validity as well as factor structure, and found good scores of validity and a moderate to good psychometric fit. With a community sample of Hispanic immigrants in Missouri, Cavazos-Rehg et al.^26^ assessed an abbreviated version of the immigrant version of the HSI and found that it maintained good internal consistency (alpha=0.86–0.87 across scales); they also measured criterion-related and construct validity as well as factor structure, and all were adequate to good fits to the data, but not content validity.

### Measures of generalized stress in Latina women

Several scales of generalized stress had one study each, which explored their psychometric properties. The Hispanic Women's Social Stressor Scale (HWSSS) was developed by Goodkind et al.^30^ by adapting the HSI to measure stress levels of Hispanic mothers in New Mexico. The 41-item scale was developed in both English and Spanish. To establish content validity, focus groups were used to confirm the most common sources of stress in Hispanic women in the southwest United States, which were divided into six subscales (each describing categories of stress experiences: acculturative stress, socioeconomic stress, racism-related stress, familial stress, parental stress, and employment stress), each of which demonstrated good to excellent internal consistency (alpha=0.73–0.94). Overall, the scale demonstrated good convergent validity with the 4-item version of the PSS. Factor structure and content, construct, and criterion-related validity were all assessed and were found to have moderate to excellent validity and reliability. The factor structure was found to be adequate.

Hannan et al.^31^ also focused on measuring stress in Hispanic women living in Florida; they translated the Life Events Inventory (LEI) and Daily Hassles Scale (DHS) into Spanish and demonstrated good internal consistency (alpha=0.94–0.97 for the LEI and alpha=0.86–0.94 for the DHS), test-retest reliability (*r*=0.86), and criterion-related validity, but did not establish content validity.

Molina et al.^33^ developed a new scale measuring stress from family obligation in Hispanic women by adapting the Caregiver Burden Scale to create the Family Obligation Stress Scale (FOSS) in English and Spanish. This FOSS is a 6-item self-report scale using a 3-item Likert scale to explore demand from and stress due to family obligation. In their study done in Washington state, this scale demonstrated fair internal consistency (alpha=0.76–0.77) and acceptable content and construct validity; factor structure was also measured and found to have a good factor structure and fit to the data.

### Other measures of generalized stress

Clinical settings were used to assess other generalized stress instruments. The Worry Scale, which asks about various sources of stress, was translated into Spanish by Novy et al.,^34^ and its psychometric properties were measured in Hispanic adults in Texas diagnosed with an anxiety-spectrum disorder. Both Spanish and English versions demonstrated excellent internal consistency in this population (alphas=0.86–0.96 and 0.89–0.97, respectively) and demonstrated acceptable criterion-related validity, but did not measure content or construct validity. Finally, the psychometric properties of the Duke Social Support and Stress Scale were measured by Katerndahl et al.^32^ by recruiting English-speaking Hispanic adults who sought care in a primary care clinic in Texas. The authors measured construct validity, which was found to be excellent, and the scale demonstrated suboptimal internal consistency for stress (alpha=0.58) and fair internal consistency for support (alpha=0.72).

## Discussion

This review reveals that since 1990 there have been eight measures of generalized stress, which have been validated in Latinos in the United States; it is critical to continue to explore the impact of stress on health and wellbeing in the largest-growing proportion of the US population that is also disproportionately impacted by high stress levels. Overall, the PSS-10 and HSI have been the most-studied, are both available in Spanish and English, and are useful to quantify and track stress. In Latina women, the HWSSS and FOSS also have potential for wider use to better understand the impact of stress. While several studies of the eight measures presented measured psychometric properties such as factor structure and internal consistency, others lacked critical aspects of survey research (e.g., content validity and criterion-related validity).^38^

The PSS-10 has demonstrated good reliability and validity in diverse populations.^37,39^ All the studies in Latinos that were captured in this review, however, failed to assess content validity and so may not fully encompass important domains of stress. Given the short length of the PSS-10 and its acceptability with diverse groups, this scale would be particularly useful in clinical or research settings involving screening or intervention.

The HSI is a much longer and more comprehensive scale, and several of the questions are much more specific to culture and ethnicity-related stress or discrimination compared to those in the PSS-10; for example, an Occupation/Economic Stress question is “Because I am Latino, it has been hard to get promotions or salary raises.” The two different versions of the full HSI (one for US-born individuals and the other for immigrants) limits its ease of use in groups that have a mixture of nationalities. Also, while this review demonstrates that the scale has been well studied in Latinos, two of the four studies utilized an abbreviated or revised version of this scale. To maintain relevance during a time of changing demographics of Latinos in the United States, the HSI was revised in 2016; the study describing the psychometric properties of the HSI-2 was not included in the current review because of its focus on acculturative stress.^40^

Two other scales described in the literature, the Hispanic Women's Social Stressor Scale (HWSSS) and the FOSS, are both useful scales to measure stressors in Latinas in the United States. They both emphasize family as an important source of stress, which is in line with prior research findings.^15^ The HWSSS, and adaptation of the HSI, could be used in groups with a blend of US-born and immigrant Latinas, but has only been studied for use with Mexican immigrants and Mexican American women. Other limitations for use of the HWSSS in clinical settings include a format, which would not lend well to self-report, and its length (interviews lasted an average of 40 min). The FOSS is a short six-item self-report, which used a 3-point Likert scale and demonstrated good content and construct validity; because of its length and self-report nature, this would be very useful to examine stress related to family roles in Latina women across clinical and research settings.

Other scales identified in this review (the Worry Scale, LEI and DHS) did not explore content or construct validity and so would need to be validated more carefully in Latino populations to improve utility in health research. The Duke Social Support and Stress Scale was studied only in English, did not have content validity established, and the stress subscale showed low internal consistency and so would be an inferior measure of stress in Latinos in the United States.

Limitations of our review include that our search was not exhaustive; it did not include all databases which publish relevant research. In addition, we did not include studies which measured psychometric properties of generalized stress scales in Latinos living in Latin America, or articles which were not written in English. Scales focusing on measuring specific types of stress, notably post-traumatic stress or acculturative stress, were not included in this review and are important sources of stress in this population.

Findings from this review reveal some critical gaps in the literature. Most of the studies have been validated in traditional immigration destinations. Literature shows that Latinos living in nontraditional immigration destinations are faced with additional challenges that may increase stress levels^41,42^ and may also have less access to the health care services necessary to address the health effects of stress compared to those who move to more traditional immigration destinations.^43,44^ As such, future research should seek to validate stress measures for these further marginalized Latino families to facilitate improved behavioral health interventions, especially in light of associations between poor mental health and the progressively more restrictive immigration policies we have observed in recent years,^45^ and the inequities highlighted by the severe acute respiratory syndrome coronavirus 2 (SARS-CoV2) pandemic.^46,47^

## Conclusion

While current evidence demonstrates that Latinos in the United States are disproportionately affected by high stress levels, only eight scales of generalized stress have had their psychometric properties studied for use in this population. Our review highlights the strengths and pitfalls of the available stress measures among Latinos in the United States and suggests uses for those that have strong validity. Future studies are warranted to assess the content validity of the PSS-10, and to assess validity in nontraditional immigration destinations that may have different demographics. Continued development and adaptation of scales to assess stress and other psychosocial constructs are important to address stress in this at-risk population.

## Abbreviations Used

CNV: content validity
CRV: criterion-related validity
CSV: construct validity
DHS: Daily Hassles Scale
FOSS: Family Obligation Stress Scale
FS: factor structure
HCHS/SOL: Hispanic Community Health Study/Study of Latinos
HSI: Hispanic Stress Inventory
HWSSS: Hispanic Women's Social Stressor Scale
IR: internal consistency
LEI: Life Events Inventory
PSS-10: 10-item Perceived Stress Scale
